# Pesticides are Associated with Allergic and Non-Allergic Wheeze among Male Farmers

**DOI:** 10.1289/EHP315

**Published:** 2016-07-06

**Authors:** Jane A. Hoppin, David M. Umbach, Stuart Long, Stephanie J. London, Paul K. Henneberger, Aaron Blair, Michael Alavanja, Laura E. Beane Freeman, Dale P. Sandler

**Affiliations:** 1 Department of Biological Sciences, and; 2 Center for Human Health and the Environment, North Carolina State University, Raleigh, North Carolina, USA; 3 Biostatistics and Computational Biology Branch, National Institute of Environmental Health Sciences (NIEHS), National Institutes of Health (NIH), Department of Health and Human Services (DHHS), Research Triangle Park, North Carolina, USA; 4 Westat, Durham, North Carolina, USA; 5 Epidemiology Branch, NIEHS, NIH, DHHS, Research Triangle Park, North Carolina, USA; 6 Division of Respiratory Disease Studies, National Institute for Occupational Safety and Health, Centers for Disease Control and Prevention, Morgantown, West Virginia, USA; 7 National Cancer Institute, NIH, DHHS, Rockville, Maryland, USA

## Abstract

**Background:**

Growing evidence suggests that pesticide use may contribute to respiratory symptoms.

**Objective:**

We evaluated the association of currently used pesticides with allergic and non-allergic wheeze among male farmers.

**Methods:**

Using the 2005–2010 interview data of the Agricultural Health Study, a prospective study of farmers in North Carolina and Iowa, we evaluated the association between allergic and non-allergic wheeze and self-reported use of 78 specific pesticides, reported by ≥ 1% of the 22,134 men interviewed. We used polytomous regression models adjusted for age, BMI, state, smoking, and current asthma, as well as for days applying pesticides and days driving diesel tractors. We defined allergic wheeze as reporting both wheeze and doctor-diagnosed hay fever (*n* = 1,310, 6%) and non-allergic wheeze as reporting wheeze but not hay fever (*n* = 3,939, 18%); men without wheeze were the referent.

**Results:**

In models evaluating current use of specific pesticides, 19 pesticides were significantly associated (*p* < 0.05) with allergic wheeze (18 positive, 1 negative) and 21 pesticides with non-allergic wheeze (19 positive, 2 negative); 11 pesticides were associated with both. Seven pesticides (herbicides: 2,4-D and simazine; insecticides: carbaryl, dimethoate, disulfoton, and zeta-cypermethrin; and fungicide pyraclostrobin) had significantly different associations for allergic and non-allergic wheeze. In exposure–response models with up to five exposure categories, we saw evidence of an exposure–response relationship for several pesticides including the commonly used herbicides 2,4-D and glyphosate, the insecticides permethrin and carbaryl, and the rodenticide warfarin.

**Conclusions:**

These results for farmers implicate several pesticides that are commonly used in agricultural and residential settings with adverse respiratory effects.

**Citation:**

Hoppin JA, Umbach DM, Long S, London SJ, Henneberger PK, Blair A, Alavanja M, Beane Freeman LE, Sandler DP. 2017. Pesticides are associated with allergic and non-allergic wheeze among male farmers. Environ Health Perspect 125:535–543; http://dx.doi.org/10.1289/EHP315

## Introduction

Worldwide, more than 5 billion pounds of pesticide active ingredients are used annually (U.S. EPA 2011a). Pesticides represent a diverse group of chemical and physical agents that have varying toxicity for both plants and animals. Pesticide exposure is common in both agricultural and residential settings; approximately 15% of the insecticides and 8% of herbicides used in the United States are for residential use. Many of the same chemicals are used in both agricultural and residential settings (U.S. EPA 2011a).

Although little is known about the potential respiratory impact of most currently used pesticides, evidence is growing that pesticide exposures contribute to respiratory symptoms and asthma. Case reports suggest that organophosphate insecticides (Bryant 1985; Weiner 1961), 2,4-D (Forbes et al. 1966), and pyrethroids (Lessenger 1992; Newton and Breslin 1983; Wagner 2000) are associated with asthma or asthmatic symptoms. Epidemiologic studies of farmers, farmworkers and commercial pesticide applicators have linked specific pesticides, including paraquat, chlorpyrifos and other organophosphates, and pyrethroids to asthma and wheeze (Castro-Gutiérrez et al. 1997; Fieten et al. 2009; Hoppin et al. 2002a, 2006, 2008, 2009; Liu et al. 2012; Ohayo-Mitoko et al. 2000; Raanan et al. 2015). Biologic mechanisms have been evaluated in animal studies for specific chemicals, including paraquat, carbaryl, and some organophosphate insecticides (Cho et al. 2008; Dong et al. 1998; Fryer et al. 2004). Large epidemiologic studies provide the opportunity to focus on specific chemicals rather than general categories of pesticide exposure such as insecticides.

To evaluate whether currently used pesticides are associated with respiratory symptoms, we used recent data from the Agricultural Health Study (AHS), a prospective study of licensed pesticide applicators and their spouses in Iowa (IA) and North Carolina (NC) (Hoppin et al. 2014). In the 2005–2010 interview, participants provided information on their current pesticide use as well as recent respiratory symptoms. Building on our earlier work (Hoppin et al. 2002a, 2006, 2008, 2009) and animal data that suggest differential effects for allergen-sensitized animals (Proskocil et al. 2008), we evaluated associations of current pesticide use with both allergic and non-allergic wheeze in male pesticide applicators. This paper includes updated analyses for 27 of the 40 pesticides previously evaluated for wheeze (Hoppin et al. 2002a, 2006) as well as initial analyses for 51 additional pesticides; chemicals with infrequent or no use during this period were not evaluated.

## Materials and Methods

### Population

We assessed pesticide exposures and wheeze among male participants in the AHS who completed the 2005–2010 follow-up interview (Hoppin et al. 2014). Participants completed a computer-aided telephone interview that collected information on current farming activities, pesticide use, medical conditions, and other demographic factors. The response rate was 51% for farmers, with the majority of nonresponse associated with our inability to contact them by phone (Hoppin et al. 2014). Questionnaires are available at http://aghealth.nih.gov/collaboration/questionnaires.html. All individuals with data on wheeze and hay fever as well as all model covariates were included. We restricted the sample to men as both the exposure profile and the risk factors differed by gender in the AHS. The AHS has been reviewed and approved by the Institutional Review Boards of the National Institues of Health (NIH) and its contractors. All participants in the AHS provided informed consent when they enrolled in the study.

### Outcome

The outcome in our analyses had three levels: no wheeze (controls), allergic wheeze, and non-allergic wheeze. This three-level outcome allowed us to assess differential response to pesticides based on allergic status. We defined a participant as having wheeze if he reported having at least one episode of wheeze or whistling in his chest in the past year. We assigned allergic status based on a history of doctor-diagnosed hay fever. Our previous work in this cohort (Hoppin et al. 2002a, 2006, 2008, 2009) used both eczema and hay fever as markers of allergy, but the current questionnaire did not collect information on eczema (and not all participants responded to the earlier questionnaire including eczema). In earlier work, hay fever alone accounted for 70% of those classified with allergic wheeze, thus our definitions are similar in this adult population. We considered individuals with both wheeze and allergy to have allergic wheeze and those with wheeze alone to have non-allergic wheeze. Controls were individuals without wheeze, irrespective of their allergy status because we were interested in allergy as a modifier of wheeze, not as an outcome.

### Exposure Assessment

Participants provided information on their current pesticide use through open-ended questions that asked about pesticide use since their last interview. Individuals provided verbatim names (usually trade names) of the pesticides that they used on crops, animals, and for non-crop purposes. These verbatim names were then linked to pesticide active ingredients’ names using the U.S. Environmental Protection Agency (EPA) Pesticide Classification Code (PC Codes; see https://www.epa.gov/ingredients-used-pesticide-products/how-search-information-about-pesticide-ingredients-and-labels) to generate a list of 515 pesticides used by participants since the last interview (Hoppin et al. 2012). Participants also provided information on the frequency (days/year) and duration (years) of use. For this analysis, we limited our evaluation to the 78 pesticides used by at least 1% of those completing the questionnaire (*n* = 22,134). Those 78 pesticides included 45 herbicides and plant growth regulators, 25 insecticides, 6 fungicides, 1 fumigant, and 1 rodenticide. A few pesticides were most often used in combination as a result of being part of the same product; therefore, there was a high degree of correlation between these chemicals, and little ability to discriminate one pesticide from the other. Notably, two pairs of herbicides had this characteristic: *a*) fenoxaprop-p-ethyl and fluazifop-butyl and *b*) clopyralid and flumetsulam. For these pairs of herbicides, we report only one set of estimates with current exposure defined as current use of either and days applied equal to the greater of the two chemicals, if they differed.

We used the current pesticide use information, along with previously reported pesticide use from the earlier interviews to create three-level pesticide use variables: current use (since the last interview), past use (not used in current cycle), and never use. We included past use as a separate category so that never users of that chemical would be the referent category, because farmers who wheezed in response to use of a specific pesticide may have stopped using that pesticide in subsequent years.

### Statistical Methods

We used polytomous regression models adjusted for current age (categories: < 50, 50–59, 60–69, 70–97 years), body mass index (BMI, < 25, 25–30, > 30), smoking status (current, past, never), current asthma (yes/no), and state (NC, IA) as well as for two current farming-related variables associated both with wheeze and pesticide use: days/year mixed or applied any pesticides (0, 1–10, 11–365 days, based on the median days of annual pesticide application) and days/year driving diesel tractors (0, < 31, 31–90, ≥ 90 days, based on questionnaire categories) to evaluate the association between pesticide exposure and allergic and non-allergic wheeze. We defined current asthma based on self-reported doctor’s diagnosis of asthma and a positive response to “Do you still have asthma?” We have used similar models in our previous analyses in this cohort (Hoppin et al. 2002a, 2006). The polytomous regression models allowed us to formally test the differences between the odds ratios (ORs) for allergic and non-allergic wheeze using a Wald test for the contrast between the two log OR parameters; a *p*-value for difference was the result of this contrast test. To assess whether correlation among pesticides could explain our findings, we assessed pairwise correlations between chemical-specific current use variables for chemicals that were significantly associated with wheeze. If the correlation exceeded 0.3, we included both chemicals in the model. All analyses were done using SAS software (version 9.3; SAS Institute Inc., Cary, NC). We used PROC LOGISTIC with the GLOGIT option for multi-level response variables and the CONTRAST statement to evaluate differences between levels.

We estimated OR for allergic and non-allergic wheeze in separate models for each chemical. Current pesticide use was parameterized in two ways: *a*) any current use and *b*) frequency of current use in categories. To create categories of use for exposure–response modeling, we divided the distribution of users into tertiles based on frequency of use; then we further subdivided the top third either in half or thirds depending on the number of users of that chemical. At a minimum, there were 10 exposed cases in each category. This modeling strategy allowed us to better evaluate the high end of the exposure distribution in this population where many individuals used chemicals for only 1 or 2 days a year.

Wheeze is the cardinal symptom of asthma and as a result most asthmatics report wheeze, though many more people report wheeze than have asthma. To assess whether inclusion of asthmatics in our models influenced our findings, we reran our models excluding those with current asthma to evaluate whether asthmatics were driving the effect estimates.

## Results

Of the 22,134 male pesticide applicators who completed the 2005–2010 interview, 1,310 (6%) had both wheeze and allergy, “allergic wheeze,” whereas 3,939 (18%) reported only wheeze “non-allergic wheeze” (Table 1). Individuals from Iowa were more likely to report non-allergic wheeze, while those in NC were more likely to report allergic wheeze. Younger farmers (< 50 years) were more likely to wheeze than older farmers. Current smokers, individuals with higher BMIs, and current asthmatics were more likely to wheeze. Although asthma was more common among those with wheeze, only 27% of those with allergic wheeze and 8% of those with non-allergic asthma reported current asthma. Farmers who applied pesticides more often and those who drove diesel tractors were more likely to report wheeze.

**Table 1:** Demographic, medical, and selected farming characteristics by wheeze status among 22,134 male pesticide applicators in the Agricultural Health Study, 2005–2010.

Characteristic	Controls *n* = 16,885 %	Allergic wheeze *n* = 1,310 %	Non-allergic wheeze *n* = 3,939 %
Age at last interview (years)
< 50	21	23	25
50–59	30	33	30
60–69	26	23	24
70–97	24	22	20
State
Iowa	67	59	70
North Carolina	33	41	30
Smoking status
Never	54	50	49
Past	39	40	37
Current	7	11	13
Current asthma	2	27	8
Hay fever diagnosis	14	100	0
BMI
< 25	20	15	16
25–30	56	51	51
> 30	24	35	33
Days/year mixed applied pesticides
None	32	28	28
1–10	37	37	38
11–365	31	35	34
Days/year drive diesel tractors
None	18	17	14
< 31	19	21	18
31–90	27	26	28
≥ 91	36	36	39

**Figure 1:**
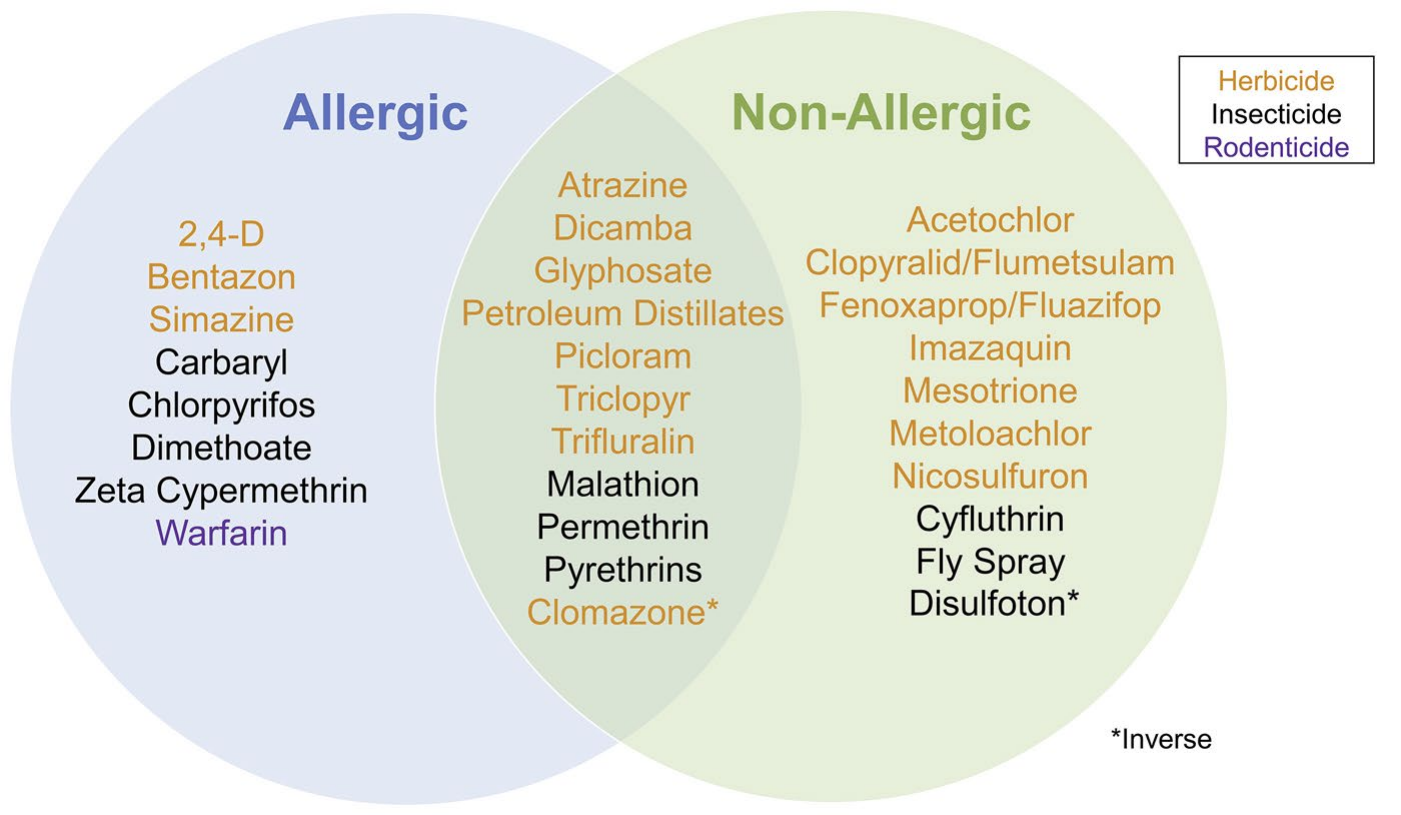
Current use pesticides associated with allergic and non-allergic wheeze among the 78 evaluated.

We evaluated current use of 78 pesticides in relation to allergic and non-allergic wheeze. Overall, 29 pesticides had some association with at least one type of wheeze, 19 were significantly associated with allergic wheeze, 21 were associated with non-allergic wheeze, and 11 pesticides were significantly associated with both. Seven had ORs for allergic and non-allergic wheeze that differed statistically (*p* < 0.05) from each other, including the commonly used herbicide 2,4-D, which had an elevated OR for allergic wheeze [OR = 1.46, 95% confidence interval (CI): 1.19, 1.79], but not for non-allergic wheeze (OR = 1.12, 95% CI: 0.99, 1.26). For ease of presentation and discussion, we have organized the results by functional group (e.g., herbicides, insecticides) in Tables 2–4. In Figure 1, we provide an overview of all the statistically significant findings from Tables 2–4. In Table 5, we present exposure–response models for 10 commonly used pesticides; all exposure–response models appear in Excel File S1.

**Table 2:** Current use of herbicides and plant growth regulators and odds ratios for allergic and non-allergic wheeze among 22,134 male participants in the Agricultural Health Study, 2005–2010.

Chemical name and group	Controls (*n* = 16,885)	Allergic wheeze (*n* = 1,310)		Non-allergic wheeze (*n* = 3,939)		*p*-Value contrast*
	Current users %	Current users %	OR (95% CI)	Current users %	OR (95% CI)	
Acetic acid herbicide
Clopyralid/flumetsulam	5	5	1.12 (0.86, 1.45)	7	1.33 (1.16, 1.54)
Dicamba	12	13	1.28 (1.04, 1.58)	15	1.29 (1.14, 1.45)
Picloram	11	12	1.28 (1.06, 1.56)	13	1.21 (1.09, 1.36)
Amide
Dimethenamid	3	3	0.91 (0.63, 1.30)	4	1.14 (0.94, 1.37)
Fomesafen	3	3	0.98 (0.68, 1.43)	3	1.17 (0.96, 1.43)
Anilide
Sulfentrazone	2	2	0.76 (0.49, 1.17)	2	0.92 (0.71, 1.20)
Benzoylcyclohexadione
Mesotrione	9	10	1.17 (0.95, 1.45)	11	1.16 (1.03, 1.31)
Chloracetanilide herbicide
Acetochlor	11	10	1.00 (0.81, 1.23)	14	1.24 (1.11, 1.39)
Alachlor	2	2	1.02 (0.68, 1.54)	2	1.25 (0.99, 1.58)
Metoachlor	11	12	1.12 (0.91, 1.37)	13	1.14 (1.01, 1.28)
Cyclohexene oxime
Clethodim	2	2	1.12 (0.76, 1.66)	2	1.00 (0.79, 1.26)
Sethoxydim	2	3	1.18 (0.81, 1.74)	2	0.80 (0.60, 1.05)	0.076
Dicarboximide
Flumioxazin	1	1	1.01 (0.54, 1.91)	1	1.03 (0.71, 1.50)
Dinitroaniline
Pendimethalin	6	7	1.07 (0.84, 1.36)	7	1.05 (0.91, 1.22)
Trifluralin	7	9	1.54 (1.22, 1.94)	9	1.24 (1.08, 1.43)	0.089
Imidazolinone
Imazapyr	1	1	0.60 (0.27, 1.33)	1	0.92 (0.62, 1.36)
Imazaquin	2	2	1.39 (0.95, 2.05)	3	1.37 (1.09, 1.71)
Imazethapyr	4	3	1.15 (0.81, 1.62)	4	1.09 (0.90, 1.32)
Nitrile
Bromoxynil	3	3	1.31 (0.93, 1.85)	3	1.12 (0.92, 1.37)
Nitrophenyl ether
Acifluorfen	1	2	1.49 (0.97, 2.28)	2	1.14 (0.85, 1.54)
Lactofen	1	1	1.53 (0.85, 2.75)	1	1.36 (0.95, 1.97)
Organophosphorous
Glufosinate ammonium	7	7	1.05 (0.82, 1.33)	9	1.11 (0.97, 1.27)
Glyphosate	56	62	1.56 (1.19, 2.03)	61	1.24 (1.07, 1.44)	0.120
Oxazole
Isoxaflutole	4	4	0.80 (0.58, 1.11)	5	0.97 (0.82, 1.15)
Plant growth regulator
Flumetralin	1	1	0.89 (0.52, 1.53)	1	0.93 (0.64, 1.35)
Maleic hydrazide	2	2	0.93 (0.62, 1.40)	2	0.89 (0.66, 1.20)
Petroleum products
Fatty alcohols	1	2	1.00 (0.61, 1.64)	1	0.97 (0.69, 1.38)
Petroleum distillates	1	2	2.45 (1.50, 4.03)	1	1.61 (1.15, 2.25)	0.123
Phenoxy herbicides
2,4-D	42	45	1.46 (1.19, 1.79)	47	1.12 (0.99, 1.26)	0.019
Fluazifop-butyl/fenoxaprop-p-ethyl	3	2	0.87 (0.58, 1.29)	4	1.25 (1.03, 1.53)	0.079
MCPP	0	0	0.89 (0.35, 2.29)	1	1.33 (0.83, 2.14)
Pyridine
Triclopyr	5	7	1.40 (1.11, 1.76)	6	1.16 (1.00, 1.35)	0.157
Quaternary ammonium
Paraquat	3	4	1.10 (0.79, 1.55)	2	0.91 (0.71, 1.16)
Sulfonanilide
Cloransulam-methyl	2	3	0.90 (0.62, 1.33)	3	1.06 (0.85, 1.31)
Triazine
Atrazine	27	28	1.33 (1.09, 1.61)	33	1.42 (1.26, 1.59)
Simazine	1	3	1.71 (1.17, 2.50)	1	0.94 (0.68, 1.28)	0.008
Triazinone
Metribuzin	1	2	1.50 (0.97, 2.30)	2	1.23 (0.93, 1.62)
Urea substitute herbicide
Chlorimuron-ethyl	2	2	1.08 (0.72, 1.64)	2	1.19 (0.93, 1.52)
Diflufenzopyr	1	1	1.28 (0.76, 2.17)	2	1.31 (0.97, 1.75)
Metsulfuron-methyl	1	1	0.79 (0.43, 1.47)	1	1.09 (0.78, 1.53)
Nicosulfuron	7	7	1.05 (0.83, 1.33)	9	1.14 (1.00, 1.30)
Rimsulfuron	4	4	1.02 (0.75, 1.40)	5	1.12 (0.94, 1.33)
Thifensulfuron-methyl	2	2	0.94 (0.59, 1.50)	2	1.15 (0.88, 1.50)
Other herbicides
Bentazon	2	3	1.56 (1.06, 2.30)	2	1.03 (0.78, 1.35)	0.058
Clomazone	1	1	0.58 (0.33, 1.00)	1	0.62 (0.43, 0.88)
Note: All models adjusted for BMI, current asthma, age, smoking status, state, days applied pesticides, and days drove diesel tractors. Referent group never used that pesticide. All pesticides were classified based on http://www.alanwood.net/pesticides. Flumetsulam is a sulfonanilide herbicide. *p-Values for contrast presented for values < 0.2.						

**Table 3:** Current use of insecticides and odds ratios for allergic and non-allergic wheeze among 22,134 male participants in the Agricultural Health Study, 2005–2010.

Chemical name and group	Controls (*n* = 16,885)	Allergic wheeze (*n* = 1,310)		Non-allergic wheeze (*n* = 3,939)		*p*-Value contrast
	Current users %	Current users %	OR (95% CI)	Current users %	OR (95% CI)	
Biological
Bacillus Thuringiensis	1	1	1.04 (0.60, 1.80)	1	0.91 (0.61, 1.34)
Carbamate
Aldicarb	1	2	1.07 (0.68, 1.69)	1	0.75 (0.52, 1.08)	0.189
Carbaryl	6	8	1.70 (1.32, 2.19)	5	1.03 (0.87, 1.22)	0.001
Carbofuran	1	1	1.19 (0.71, 2.00)	1	0.98 (0.70, 1.36)
Neonicotinoid
Imidacloprid	1	1	0.78 (0.44, 1.38)	1	1.27 (0.91, 1.76)	0.118
Organochlorine
Endosulfan	1	1	1.01 (0.57, 1.79)	1	0.89 (0.59, 1.35)
Lindane	1	1	1.58 (0.93, 2.70)	1	0.93 (0.63, 1.38)	0.087
Organophosphorous
Acephate	5	6	0.84 (0.64, 1.10)	5	0.85 (0.70, 1.02)
Chlorpyrifos	9	11	1.23 (1.00, 1.52)	10	1.09 (0.96, 1.24)
Diazinon	2	2	1.31 (0.86, 1.98)	2	0.93 (0.70, 1.24)	0.151
Dimethoate	1	2	1.67 (1.03, 2.73)	1	0.73 (0.46, 1.15)	0.008
Disulfoton	1	2	1.17 (0.73, 1.87)	1	0.63 (0.42, 0.95)	0.037
Malathion	11	12	1.48 (1.19, 1.86)	13	1.29 (1.13, 1.46)
Phosmet	2	2	1.21 (0.78, 1.90)	2	1.26 (0.97, 1.63)
Tebupirimfos	5	4	0.85 (0.63, 1.15)	6	1.13 (0.97, 1.31)	0.082
Terbufos	2	3	1.16 (0.81, 1.68)	3	1.10 (0.87, 1.37)
Pyrethroid
Bifenthrin	1	1	0.99 (0.54, 1.78)	1	1.06 (0.75, 1.51)
Cyfluthrin	8	8	1.02 (0.81, 1.28)	9	1.13 (1.00, 1.29)
Esfenvalerate	2	2	0.94 (0.61, 1.46)	2	0.99 (0.76, 1.31)
Lambda Cyhalothrin	4	5	1.19 (0.89, 1.59)	4	1.05 (0.88, 1.26)
Permethrin	6	7	1.38 (1.09, 1.75)	8	1.35 (1.17, 1.55)
Pyrethins	1	2	1.70 (1.13, 2.56)	2	1.43 (1.10, 1.85)
Tefluthrin	3	3	1.09 (0.78, 1.52)	4	1.03 (0.86, 1.25)
Zeta Cypermethrin	1	2	2.02 (1.24, 3.30)	1	0.88 (0.60, 1.30)	0.005
Other
Fly spray	1	2	1.17 (0.72, 1.89)	2	1.43 (1.10, 1.86)
Note: All models adjusted for BMI, current asthma, age, smoking status, state, days applied pesticides, and days drove diesel tractors. Referent group never used that chemical. All pesticides were classified based on http://www.alanwood.net/pesticides. *p*-Values for contrast presented for *p* < 0.2.						

**Table 4:** Current use of fumigants, fungicides, and rodenticides and odds ratios for allergic and non-allergic wheeze among 22,134 male participants in the Agricultural Health Study, 2005–2010.

Chemical name and group	Controls (*n* = 16,885)	Allergic wheeze (*n* = 1,310)		Non-allergic wheeze (*n* = 3,939)		*p*-Value contrast
	Current users %	Current users %	OR (95% CI)	Current users %	OR (95% CI)	
Fumigant
Chloropicrin	1	2	1.00 (0.60, 1.67)	1	0.93 (0.64, 1.34)
Fungicides
Captan	2	2	1.20 (0.80, 1.81)	2	1.00 (0.76, 1.32)
Chlorothalonil	2	2	0.96 (0.64, 1.43)	2	0.81 (0.61, 1.08)
Mancozeb	1	1	0.93 (0.52, 1.68)	1	1.11 (0.77, 1.61)
Metalaxyl	2	2	0.74 (0.46, 1.18)	1	0.84 (0.63, 1.14)
Propioconazole	1	1	0.96 (0.52, 1.79)	1	0.68 (0.43, 1.06)
Pyraclostrobin	2	3	1.46 (0.99, 2.14)	2	0.94 (0.72, 1.23)	0.045
Rodenticide
Warfarin	2	2	1.55 (1.04, 2.30)	2	1.26 (0.98, 1.62)
Note: All models adjusted for BMI, current asthma, age, smoking status, state, days applied pesticides, and days drove diesel tractors. Referent group never used that pesticide. All pesticides were classified based on http://www.alanwood.net/pesticides. *p*-Value for contrast presented for values < 0.2.						

**Table 5:** Selected exposure–response models for pesticides and allergic and non-allergic wheeze among 22,134 male farmers in the Agricultural Health Study (2005–2010).

Chemical	Days/year used	Controls (*n* = 16,885)	Allergic wheeze (*n* = 1,310)		Non-allergic wheeze (*n* = 3,939)	
		Current users %	Current users %	OR (95% CI)	Current users %	OR (95% CI)
Herbicides
2,4-D	Never	17	15	1.00	16	1.00
	Past	40	39	1.23 (1.03, 1.48)	37	0.96 (0.86, 1.07)
	1–3	16	16	1.34 (1.06, 1.71)	17	1.10 (0.96, 1.27)
	4–7	13	13	1.41 (1.10, 1.81)	14	1.11 (0.96, 1.29)
	8–10	6	6	1.35 (0.99, 1.82)	7	1.07 (0.90, 1.28)
	11–15	3	4	1.84 (1.30, 2.60)	3	1.08 (0.87, 1.35)
	16–365	4	6	1.87 (1.38, 2.55)	6	1.29 (1.07, 1.55)
Atrazine	Never	23	22	1.00	19	1.00
	Past	50	50	1.29 (1.10, 1.52)	48	1.15 (1.04, 1.27)
	1–4	10	9	1.20 (0.94, 1.54)	12	1.41 (1.23, 1.63)
	5–7	8	9	1.47 (1.13, 1.90)	9	1.35 (1.15, 1.58)
	8–10	5	5	1.40 (1.03, 1.91)	6	1.50 (1.26, 1.80)
	11–14	1	2	1.81 (1.11, 2.94)	2	1.72 (1.28, 2.31)
	15–122	3	3	1.20 (0.83, 1.74)	4	1.39 (1.12, 1.73)
Dicamba	Never	46	48	1.00	42	1.00
	Past	42	39	1.13 (0.97, 1.32)	44	1.11 (1.01, 1.21)
	1–2	3	3	1.09 (0.76, 1.56)	4	1.25 (1.02, 1.52)
	3–5	5	5	1.30 (0.97, 1.75)	6	1.22 (1.03, 1.45)
	6–7	1	1	1.20 (0.70, 2.06)	2	1.44 (1.08, 1.92)
	8–10	1	1	1.12 (0.66, 1.90)	2	1.42 (1.08, 1.87)
	11–110	1	2	2.00 (1.27, 3.16)	1	1.35 (0.99, 1.85)
Glyphosate	Never	11	8	1.00	9	1.00
	Past	33	30	1.13 (0.90, 1.43)	29	1.05 (0.92, 1.19)
	1–5	20	21	1.47 (1.11, 1.96)	22	1.23 (1.05, 1.44)
	6–11	16	17	1.57 (1.17, 2.10)	18	1.24 (1.05, 1.46)
	12–15	6	8	1.73 (1.24, 2.44)	8	1.34 (1.10, 1.63)
	16–25	7	8	1.62 (1.15, 2.28)	8	1.24 (1.02, 1.52)
	25–240	6	8	1.79 (1.26, 2.53)	7	1.23 (1.00, 1.51)
Insecticides
Carbaryl	Never	41	32	1.00	41	1.00
	Past	53	61	1.48 (1.29, 1.69)	54	1.08 (1.00, 1.16)
	1–2	2	2	1.45 (0.95, 2.20)	2	1.02 (0.78, 1.34)
	3–6	2	3	2.07 (1.43, 3.01)	2	1.12 (0.86, 1.47)
	7–10	1	1	1.24 (0.71, 2.17)	1	1.00 (0.69, 1.44)
	11–100	1	1	1.55 (0.87, 2.76)	1	0.76 (0.48, 1.20)
Chlorpyrifos	Never	54	51	1.00	53	1.00
	Past	37	39	1.13 (0.99, 1.28)	37	0.98 (0.91, 1.06)
	1–2	3	3	1.24 (0.87, 1.76)	3	1.05 (0.85, 1.31)
	3–5	3	4	1.12 (0.81, 1.55)	4	1.12 (0.93, 1.35)
	6–8	1	2	1.48 (0.90, 2.45)	1	0.92 (0.65, 1.31)
	9–10	1	1	1.17 (0.64, 2.12)	1	1.31 (0.94, 1.82)
	11–120	1	1	1.29 (0.72, 2.32)	1	1.07 (0.73, 1.55)
Cyfluthrin	Never	91	91	1.00	89	1.00
	Past	1	1	0.88 (0.51, 1.52)	2	1.19 (0.90, 1.58)
	1–3	3	3	0.99 (0.68, 1.43)	3	1.04 (0.84, 1.29)
	4–6	2	3	1.16 (0.80, 1.68)	3	1.13 (0.90, 1.41)
	7–9	1	1	0.96 (0.51, 1.82)	1	1.25 (0.90, 1.74)
	10–11	1	1	0.98 (0.50, 1.91)	1	1.42 (1.02, 1.98)
	12–200	1	1	0.79 (0.40, 1.54)	1	1.08 (0.76, 1.53)
Malathion	Never	26	22	1.00	24	1.00
	Past	63	67	1.39 (1.20, 1.61)	63	1.09 (1.00, 1.19)
	1	4	3	0.99 (0.70, 1.42)	5	1.19 (1.00, 1.43)
	2	2	4	1.93 (1.36, 2.75)	3	1.32 (1.06, 1.64)
	3–4	1	2	2.00 (1.30, 3.08)	2	1.34 (1.01, 1.77)
	5–7	1	2	1.85 (1.12, 3.04)	1	1.18 (0.85, 1.64)
	8–100	1	1	1.08 (0.60, 1.95)	2	1.57 (1.16, 2.12)
Permethrin	Never	73	69	1.00	70	1.00
	Past	22	24	1.19 (1.03, 1.38)	22	1.05 (0.96, 1.14)
	1–2	2	2	1.31 (0.88, 1.95)	3	1.19 (0.94, 1.50)
	3–6	2	3	1.42 (0.97, 2.09)	3	1.29 (1.02, 1.63)
	7–12	1	1	1.18 (0.67, 2.08)	1	1.52 (1.12, 2.08)
	13–365	1	1	1.79 (1.05, 3.04)	2	1.76 (1.30, 2.39)
Rodenticide
Warfarin	Never	97	96	1.00	96	1.00
	Past	1	2	1.53 (0.99, 2.38)	2	1.33 (1.02, 1.72)
	1–2	0	1	1.50 (0.75, 3.01)	1	1.06 (0.65, 1.71)
	3–6	1	1	1.40 (0.71, 2.74)	1	1.56 (1.07, 2.29)
	7–260	1	1	1.81 (0.92, 3.56)	1	1.07 (0.68, 1.71)
Note: All models adjusted for BMI, current asthma, age, smoking status, state, days applied pesticides, and days drove diesel tractors. Referent group never used that chemical. Categories are tertiles of the exposure distribution with the top tertile cut in half or thirds, depending on frequency of use of chemical in the past year. Some percentages do not sum to 100 due to rounding.						

### Herbicides and Plant Growth Regulators

Of the 43 herbicides and two plant growth regulators, 18 were associated with at least one wheeze outcome (Table 2). Only one was inversely associated with wheeze, and it was inversely associated with both allergic and non-allergic wheeze (clomazone). Fourteen herbicides were positively associated with non-allergic wheeze and 10 with allergic wheeze. Three herbicides were associated only with allergic wheeze, while seven herbicides were associated only with non-allergic wheeze. Two herbicides (2,4-D and simazine) had statistically significant contrasts (*p* < 0.05) and were positively associated only with allergic wheeze.

Wheeze prevalence differed by herbicide chemical group. All three acetic acid herbicides (clopyralid/flumetsulam, picloram, and dicamba) were significantly associated with non-allergic wheeze and both picloram and dicamba were associated with allergic wheeze as well. Two of the three chloracetanilide herbicides (acetochlor and metolachlor) were significantly associated with non-allergic wheeze, and the third alachlor had an elevated but non-significant OR for non-allergic wheeze (1.25, 95% CI: 0.99, 1.58); there was no evidence of an association with allergic wheeze for this class of herbicides. Of the two dinitroaniline herbicides, only trifluralin was associated with non-allergic and allergic wheeze. Pendimethalin was not associated with wheeze. Of the three imidazolinone herbicides, only imazaquin was associated with wheeze and only significantly with non-allergic wheeze. Glyphosate, the most commonly used herbicide, was significantly associated with both types of wheeze, while the related but less commonly used glufosinate ammonium was not associated with either wheeze outcome. Of the three phenoxy herbicides, 2,4-D was significantly associated with allergic wheeze, while the fluazifop-butyl/fenoxaprop-p-ethyl was associated with non-allergic wheeze. The two triazines (atrazine, simazine) were significantly associated with allergic wheeze, while the related metribuzin (a triazinone) also had an elevated but not statistically significant association with allergic wheeze. Atrazine, but not simazine or metribuzin, was also associated with non-allergic wheeze. Use of petroleum distillates had the highest odds of wheeze for both non-allergic (OR = 1.61, 95% CI: 1.15, 2.25) and allergic wheeze (OR = 2.45, 95% CI: 1.50, 4.03).

### Insecticides

Current use of nine of the 25 individual insecticides was positively associated with at least one type of wheeze, and one insecticide (disulfoton) was inversely associated with non-allergic wheeze (Table 3). Two of the pyrethroids (permethrin, pyrethrins) were significantly associated with both allergic and non-allergic wheeze, while a third, zeta-cypermethrin was strongly associated with allergic wheeze (OR = 2.02, 95% CI: 1.24, 3.30) and inversely, but not significantly so, with non-allergic wheeze (OR = 0.88, 95% CI: 0.60, 1.30; *p*_contrast_ = 0.005). Of the organophosphates, malathion was associated with both allergic and non-allergic wheeze, while chlorpyrifos and dimethoate were associated only with allergic wheeze. Carbaryl, a carbamate insecticide, was associated with allergic (OR = 1.70, 95% CI: 1.32, 2.19), but not non-allergic wheeze (OR = 1.03, 95% CI: 0.87, 1.22; *p*_contrast_ = 0.001). Fly spray use was associated with non-allergic wheeze.

### Fungicides, Fumigant, and Rodenticide

In addition to herbicides and insecticides, we evaluated six fungicides, one fumigant, and one rodenticide for association with wheeze (Table 4). Of these, only the rodenticide warfarin was significantly associated with allergic wheeze (OR = 1.55, 95% CI: 1.04, 2.30), none of the chemicals were associated with non-allergic wheeze. Additionally, while not statistically significant, the fungicide pyraclostrobin was positively associated with allergic wheeze (OR = 1.46, 95% CI: 0.99, 2.14) but not with non-allergic wheeze (OR = 0.94, 95% CI: 0.72, 1.23; *p*_contrast_ = 0.045).

### Correlated Pesticides

Because pesticides can be used in combination or on the same crop over the course of a growing season, we evaluated whether correlation among the pesticides were responsible for the large number of significant findings. Among the pesticides associated with at least one category of wheeze, there were 27 pairs of Spearman correlations for pesticides that exceeded 0.3. Twelve pesticides (nine herbicides: 2,4-D; acetochlor, atrazine, dicamba, glyphosate, mesotrione, metolachlor, nicosulfuron, trifluralin; three insecticides: dimethoate, disulfoton, and malathion) contributed to these 27 pairs. When we included the correlated pairs of pesticides in the same model, we saw no strong evidence of confounding with only small changes in the ORs for the individual chemicals (data not shown).

### Exposure–Response Modeling

We constructed exposure–response models for all pesticides. Ten chemicals (seven herbicides, three insecticides) had sufficient numbers of frequent users to create five-level exposure variables where the top third was itself split into thirds. Nine chemicals had four categories of exposure; the remaining 59 were split at tertiles of current use (see Excel File S1). Table 5 presents exposure–response models for 10 of the 78 pesticides, which we chose based on the frequency of use and evidence of an association with the outcome. The herbicides 2,4-D and glyphosate both showed higher prevalence of allergic wheeze with increasing use. While both also showed an association with non-allergic wheeze, the association for 2,4-D was only present among those who used 2,4-D at least 16 days/year. Atrazine had similar exposure–response profiles for both allergic and non-allergic wheeze, with increasing ORs up to 14 days per year and with a lower OR in the highest exposure category. Among the insecticides, carbaryl showed the greatest difference between allergic and non-allergic wheeze, with any level of use associated with increased allergic wheeze, but not with increased non-allergic wheeze. The organophosphates malathion and chlorpyrifos both showed elevated odds of allergic wheeze, but there was no strong evidence of an exposure–response relationship. Use of permethrin was associated with allergic and non-allergic wheeze with the highest odds associated with the highest level of use (13–365 days). The rodenticide warfarin was associated with increased allergic wheeze with increasing use.

When we limited the analysis to those without current asthma, we observed essentially the same findings for both allergic and non-allergic wheeze. Seven pesticides (herbicides: 2,4-D; acetochlor; and simazine; insecticides: carbaryl, tebupirimfos, dimethoate, zeta-permethrin) had significantly different estimates for allergic and non-allergic wheeze, but the estimates were similar to those for the whole sample (data not shown).

## Discussion

Our study evaluated a more comprehensive list of currently used pesticides in relation to wheeze than has ever been evaluated for any other respiratory outcome. In our sample of 22,134 male farmers from the AHS cohort, we included 78 currently used individual pesticides, 51 of which had not been previously analyzed for respiratory outcomes. These pesticides included some used solely for agricultural purposes (e.g., paraquat) as well as some with residential and public health uses in addition to agriculture (e.g., glyphosate, 2,4-D, permethrin). We also considered allergic and non-allergic wheeze separately because these outcomes may have different etiologies; our previous analyses may have masked associations with allergic wheeze given its lower prevalence. Overall, 29 pesticides had some association with at least one type of wheeze; 19 were significantly associated with allergic wheeze and 21were associated with non-allergic wheeze; 11 pesticides were significantly associated with both. These associations remained when we excluded asthmatics and when we adjusted for correlated pesticides. Exposure–response analyses provided additional evidence for some pesticides, although many of these chemicals were used infrequently making it difficult to evaluate a quantitative relationship. While non-allergic wheeze was three times more common than allergic wheeze, the associations with pesticides were generally of greater magnitude with allergic wheeze. This observation could suggest that individuals with allergic wheeze are more responsive to the environment; which is consistent with animal models that suggest the respiratory impact of some pesticides is stronger in those with allergy (Dong et al. 1998; Proskocil et al. 2008).

In comparing these cross-sectional results to our previous cross-sectional work among farmers (Hoppin et al. 2002a) and commercial pesticide applicators (Hoppin et al. 2006), we see generally similar findings for the chemicals included in these analyses. This sample is similar, but not identical, to our 2002 analysis: 56% (*n* = 12,331) of the current participants provided information on wheeze at enrollment, while 44% did not. It is possible that some individuals most affected by pesticides chose not to complete the most recent interview, however, it is unlikely to influence our overall findings, and in fact, the prevalence of wheeze is slightly higher in the current analysis (24%) than in the 2002 analysis (21%). Furthermore, this analysis, as was the first, is cross-sectional based on current exposures and current wheeze, so loss to follow-up due to pesticide use at enrollment is not likely to have an impact on the validity of these results, although generalizability to the entire cohort may be affected if persons who were more sensitive to pesticides were more likely to have dropped out.

From the first analysis in 2002 to the current analysis, the prevalence of use of many of the chemicals has decreased. Only glyphosate use increased from the 1993–1997 time frame. Of the 16 herbicides that we evaluated previously, three [butylate, cyanazine, and *S*-ethyl dipropylthiocarbamate (EPTC)] were not evaluated here due to infrequent use. The largest difference between the 2002 paper (Hoppin et al. 2002a) and the current analysis, is the strong positive findings here for 2,4-D and allergic wheeze. When we previously analyzed wheeze as a single outcome, we saw no association for 2,4-D and wheeze, but when we stratified on allergic status, we saw an association that was limited to allergic wheeze as well as evidence of an exposure–response relationship. We also saw associations of dicamba with both allergic and non-allergic wheeze in the current analysis that we did not see for wheeze overall previously. We continued to see associations with atrazine, glyphosate, trifluralin, and petroleum oil. We no longer observed an association with paraquat; a chemical for which use has declined over the 10-year period of AHS data collection. For insecticides, we evaluated nine of the previous 15, and continued to see associations for permethrin, carbaryl, and malathion. We previously observed associations with chlorpyrifos use, particularly among commercial pesticide applicators (Hoppin et al. 2006), that we did not see here, but frequency of chlorpyrifos has decreased dramatically since 1993–1997. At that time, 38% of the cohort was applying chlorpyrifos 5 or more days a year; for this analysis, only 3% were applying 6 or more days/year (the top third). None of the fungicides that we evaluated previously were associated with wheeze earlier or in the current analysis.

The three most commonly used herbicides—glyphosate, 2,4-D, and atrazine—were associated with wheeze with evidence of an exposure–response relationship for at least one of the wheeze outcomes; correlations among these pesticides did not explain these findings. Glyphosate and atrazine were associated with both allergic and non-allergic wheeze, whereas 2,4-D was primarily associated with allergic wheeze. Use of glyphosate, commonly sold under the trade name Roundup^®^, has increased dramatically since our initial analysis due to the widespread use of Roundup^®^ Ready corn and soybean seed. The percentage of farmers applying glyphosate 20 or more days/year increased from 5% to 11% between enrollment (1993–1997) and the current interview (2005–2010). With this increased frequency of use, we had more power to detect associations; but the effect estimates were very similar to those reported previously. Animal studies have suggested a potential mechanism for glyphosate-induced airway inflammation; glyphosate exposures increased eosinophil and neutrophil counts, mast cell degranulation, and production of the cytokines IL-33, TSLP, IL-13, and IL-5; co-administration of ovalbumin did not change the inflammatory immune response (Kumar et al. 2014). Glyphosate was also associated with rhinitis among both farmers and commercial applicators in the AHS (Slager et al. 2009, 2010). In commercial applicators, 2,4-D was associated with rhinitis only when used with glyphosate; we did not see similar evidence of an interaction for either type of wheeze, but the farmers evaluated here use chemicals much less frequently than do commercial applicators. In asthma analyses in the AHS, 2,4-D was significantly associated with allergic, but not non-allergic asthma among women (Hoppin et al. 2008), and with a monotonic increase in allergic asthma prevalence among male farmers (Hoppin et al. 2009). In animal studies, 2,4-D has been associated with respiratory allergy in mice (Cushman and Street 1982) and with sensitization and subsequent respiratory IgE allergic response in mice (Fukuyama et al. 2009). While we continued to see an association of wheeze with atrazine use, animal data to evaluate this finding are limited. In our earlier analysis of commercial pesticide applicators (Hoppin et al. 2006), the association between atrazine and wheeze was confounded by chlorimuron-ethyl, a chemical not commonly used by farmers.

Organophosphate and carbamate insecticides have been associated with wheeze and asthma in this (Hoppin et al. 2002a, 2006, 2008, 2009) and other populations (Fieten et al. 2009; Raanan et al. 2015; Senthilselvan et al. 1992; Yemaneberhan et al. 1997). Here, we saw diminished evidence for an association between respiratory symptoms and use of organophosphate insecticides, although malathion continued to be associated both with allergic and non-allergic wheeze. Malathion was associated with increased skin-prick test sensitization to *Dermatophagoides pteronyssinus* in an Ethiopian study (Yemaneberhan et al. 1997). Chlorpyrifos was associated with allergic wheeze with increased use, but the exposure–response results were not strong. In animal studies, organophosphate insecticides contribute to airway hypersensitivity through the decreasing M2 muscarinic receptor responsiveness and not through acetylcholinesterase inhibition (Fryer et al. 2004); moreover, allergen-sensitized animals are more responsive (Proskocil et al. 2008). To date, animal models have been used to evaluate parathion, diazinon, and chlorpyrifos; whether malathion may also act through these mechanisms is not known.

Carbaryl, a carbamate insecticide sold under the trade name Sevin, was significantly associated with allergic wheeze with strong evidence of an exposure–response relationship. Carbaryl was the third most commonly used pesticide in the home and garden sector and the most commonly used insecticide in 2007 (U.S. EPA 2011a). Carbamate insecticides as a group, and carbofuran in particular, were associated with self-reported asthma among farmers in Saskatchewan, Canada (Senthilselvan et al. 1992). In rats, carbaryl enhanced the pulmonary allergic responsiveness to house dust mite (Dong et al. 1998) and has been shown to alter the Th1/Th2 balance in developing rats (Jorsaraei et al. 2014).

Pyrethroids are among the most commonly used insecticides, particularly for residential uses. In 2007, pyrethroids as a group were the second most commonly used insecticide in home and garden settings (U.S. EPA 2011a). All the pyrethroids that we evaluated, except tefluthrin, are approved for residential uses (U.S. EPA 2011b). Three of the eight pyrethroid insecticides were associated with wheeze. Cyfluthrin, the most commonly used pyrethroid in this study, was not. The most frequent users of permethrin were more likely to report wheeze, both allergic and non-allergic. Other investigators have found suggestive evidence of wheeze associated with permethrin exposure among children (Liu et al. 2012). Pyrethroids as a group may influence production of the cytokine IL-10 (Neta et al. 2011) and interferon-gamma and IL-4 (Diel et al. 2003) in humans, but studies are limited. In 2009, the U.S. Environmental Protection Agency (EPA) reviewed the relationship between pyrethroid exposure and asthma and allergy and found “no clear or consistent pattern of effects reported to indicate conclusively whether there is an association between pyrethrins/pyrethroid exposure and asthma and allergy;” however, all pesticide registrants were asked to provide the U.S. EPA with detailed follow-up of reported pyrethrins incident cases (U.S. EPA 2009).

The rodenticide warfarin was strongly associated with increased allergic wheeze. Some of this increase is possibly due to the environments in which rodenticides are used on farms (barns, silos) which may result in increased allergen exposure. Warfarin is also a commonly used anti-coagulation agent in human medicine. Wheezing is one of the rarer reported side effects (http://www.drugs.com/sfx/warfarin-side-effects.html) and allergic reactions are considered an even more serious side effect.

In conducting this analysis, we did not adjust for multiple comparisons nor did we undertake analysis of potential mixtures. Other authors (Goldberg and Silbergeld 2011; Rothman 1990) have argued against adjusting for multiple comparisons with preference for data presentation consistent with Bradford–Hill criteria (Hill 1965), where study design, analytical approach, and consistency of the evidence over time or across study populations are given priority. Even in a sample as large as ours, analysis of potential combinations of agents is challenging, particularly when most chemicals are used fewer than 5 days a year. Overall the effect estimates were generally low (ORs ranging 1.13–2.45) which is an advantage of our large sample size, but suggests that residual confounding may explain our associations. We did use statistical measures to minimize potential confounding by including a covariate for overall pesticide use in the growing seasons and frequency of driving diesel tractors. We grouped pesticides that came from the same technical product, as neither we nor the users are able to separate out exposure to the individual chemicals. We saw more statistical associations than would be expected by chance (5% of 156 comparisons equals eight expected significant chemicals where we observed 40 significant); and, for a few chemicals, limited laboratory evidence supports these associations. The lack of comprehensive toxicological testing for respiratory and allergic hazards presents a challenge in interpreting our findings. One issue in interpretation is the lack of ability to evaluate the other ingredients in these pesticides because this information is not publicly available (U.S. EPA 2003; Petrelli et al. 1993). It is possible that some common other ingredient could explain these associations for specific pesticides, but we are not able to evaluate that using our data. The fact that associations differed by pesticide argues that systematic bias does not explain these results.

Evaluating the respiratory outcomes associated with pesticides is challenging and requires a large number of individuals who are able to provide detailed information regarding their personal pesticide usage. The AHS has both these strengths. Pesticide use history in the AHS is detailed and has been demonstrated to be reproducible (Blair et al. 2002) and accurate (Hoppin et al. 2002b). We evaluated the common respiratory symptom, wheeze, which by nature is a self-reported outcome. Questionnaire assessment of wheeze has been demonstrated to be reliable and reproducible (Burney et al. 1989). Because our study relied on self-reported symptom and pesticide use in the same interview cycle, we cannot be certain that pesticide use preceded respiratory symptoms; this feature also limited our exposure–response analysis. By restricting our investigation to currently used pesticides, we have done our best to ensure that these pesticides were used in roughly the same time window as when wheeze was assessed. It is unlikely that recall bias influenced these findings; not all pesticides were associated with wheeze and we observed differential associations for allergic and non-allergic wheeze. Even if farmers were aware of our previous findings, recall bias could not explain the differential findings for allergic and non-allergic wheeze or the findings for some, but not all, new chemicals. When we limited our analysis to participants without asthma, we saw essentially the same results. Because people may stop using a chemical that triggers respiratory symptoms, we created a separate category for past use of the chemical, so that the referent group was never users of the chemical. For some chemicals, past users had higher odds of wheeze than never users. However, we do not have the data to evaluate whether this observation is an indication of a lingering impact of use or some other exposure. For every chemical, current users at some exposure level had ORs higher than former users.

This is the most comprehensive analysis of current use pesticides and the common respiratory symptom wheeze to date. Our analysis included the majority of pesticides used in agriculture, home and garden, and industrial/commercial/governmental uses in the United States (U.S. EPA 2011a). Our sample includes nine of the 10 most commonly used pesticides in the home and garden sector; six of 10 in the industry/commercial/government sector; and 16 of the 25 in the agricultural market sector (U.S. EPA 2011a). Therefore, our analysis is a good representation of the pesticides used in the United States. While this analysis was limited to male farmers who, most likely, have applied pesticides for decades, the chemicals that they use are not exclusively agricultural. The findings for chemicals like glyphosate, 2,4-D, carbaryl, and the pyrethroids are particularly relevant for consumers who would like to minimize their wheeze and allergy risk associated with the use of chemicals in their homes, gardens and play areas. While 29 of the 78 pesticides showed some association with wheeze, the majority did not. Future studies should focus on potential mechanisms as well as strategies to minimize exposure.

## Supplemental Material

(63 KB) ZIP
